# Recycled PET/PA6 Fibers from Waste Textile with Improved Hydrophilicity by In-Situ Reaction-Induced Capacity Enhancement

**DOI:** 10.3390/polym16081052

**Published:** 2024-04-11

**Authors:** Li-Bin Luo, Rong Chen, Yu-Xin Lian, Wen-Jun Wu, Jia-Hong Zhang, Chong-Xian Fu, Xiao-Li Sun, Li-Ren Xiao

**Affiliations:** 1 Engineering Research Center of Polymer Green Recycling of Ministry of Education, Fujian Normal University, Fuzhou 350007, China; qsz20211438@student.fjnu.edu.cn (L.-B.L.); 18559387800@163.com (R.C.); 18150639551@163.com (Y.-X.L.); 17805931286@163.com (W.-J.W.); 2 College of Chemistry and Materials Science, Fujian Normal University, Fuzhou 350007, China; 3 College of Environmental and Resource Science, College of Carbon Neutral Modern Industry, Fujian Normal University, Fuzhou 350007, China; 4 Fujian Baichuan Resource Recovery Technology Co., Ltd., Quanzhou 362100, China; kaihung@fjbaichuan.com; 5 Fujian Eversun Jinjiang Co., Ltd., Fuzhou 350200, China; fuchongxian@126.com

**Keywords:** waste textile, polyester, polyamide, mechanical recycling, rheological property

## Abstract

Due to the increasing amounts of textile waste, textile to textile recycling is of prime concern. Polyethylene terephthalate (PET) represents the most extensively used type of chemical fiber. Its spinnability suffers from impurities and degradation in the processing, which limits its recycling to new fibers. Here, recycled polyester is blended with a small amount of recycled nylon, and the regenerated fibers, which demonstrated good mechanical properties, were obtained via a melt spinning machine. The mechanical properties, thermal properties, rheological properties, and chemical structure of the modified recycled fibers were investigated. It was found that when compared with rPET-T fibers, the elongation at break of rPET-A_x_ fibers increased to 17.48%, and the strength at break decreased to 3.79 cN/dtex. The compatibility of PET and PA6 copolymer were enhanced by copolymers produced by in-situ reaction in the processing. Meanwhile, the existence of PA6 increases the crystallization temperature and improves the hydrophilicity of the fibers. This study realized the high-value recycling of waste PET fabric to new fibers, which opens a door for the large utilization of waste textiles.

## 1. Introduction

Encouraged consumption of textile products and rapidly changing fashion trends have generated tremendous amounts of textile waste worldwide [1,2,3,4,5]. The dominant treatment was incineration and landfills, which causes environmental problems. An increasing tendency to recycle textile waste was observed [6], and textile to textile recycling is of prime concern.

Textile waste is composed of a variety of substances, including natural as well as synthetic fibers, which makes textile to textile recycling challenging. Among them, polyethylene terephthalate (PET) fibers account for more than 80% of global fiber production [7,8,9]. There have been several studies on textile to textile recycling of waste PET fabric, including studies on chemical recovery and physical recovery [10,11,12,13,14]. Through chemical recovery methods such as alcoholysis, hydrolysis, and ammonolysis, high quality regenerated fibers are obtained via repolymerization of the intermediate product, including dimethyl terephthalate (DMT), dihydroxyethyl terephthalate (BHET), or 1,4-diphenyl terephthalate (TPA). However, its high cost, cumbersome process, and necessary pollution control in the recovery process significantly hinders its industrial application.

PA6 is also widely used in the textile industry because of its high wear resistance, high strength, and corrosion resistance, and it has become another kind of widely used chemical fiber. The waste textile often contains PET and PA6. However, compatibility has always been a key research issue in the blending of PET and PA6 [15,16]. Compatibility is usually improved by adding a compatibilizer to increase the adhesion of the two-phase interface. Huang improved the compatibility of PET/PA6 blends by adding epoxy resin E-44, which reduced the size of the dispersed phase, and increased the impact strength and bending strength of the blends by 500% and 400%, respectively [17]. The interaction between PET and PA6 can be changed by adding ethylene-acrylate-maleic anhydride (E-AE-MAH), and the impact strength of the blend is increased from 2.3 kJ/m^2^ to 5.0 kJ/m^2^ [18]. However, it is found that PET/PA6 blends spontaneously undergo an ester-amide exchange reaction during melt mixing, resulting in the production of a copolymer, which can increase compatibility. TsOH has been proved to be an efficient catalyst for the ester-amide exchange reaction, which can effectively improve the two-phase compatibility and the mechanical properties of the blends, but TsOH is not suitable for the recovery method with a short processing process [19]. The compatibilization or modification of the PET/PA6 blend system can be realized in different ways, but the modified blend is difficult to be use in fiber production.

In this work, a low proportion (≤10 wt%) of regenerated polyamide particles were blended with recycled polyester from waste fabric to improve its spinning property. Polyamide particles were used not only because they exist in the waste textile as the second highest-ranked synthetic fiber, but also because of its potential to improve the hydrophilicity of polyester. The compatibility of PET/PA6 in the blending system was successfully enhanced by in-situ reaction during the processing, and recycled fibers were obtained. This study opens a door for utilization of waste PET textiles in textile to textile recycling.

## 2. Materials and Methods

### 2.1. Materials

Waste fiber source polyester pellets (rPET−F) were provided by Fujian Baichuan Resource Recovery Technology Co., Ltd. (Quanzhou, China). These pellets are fabricated by small scraps of waste polyester fabric being passed through granulation. The intrinsic viscosity of the rPET−F was measured in the range of 0.52–0.57 dL/g, the melt mass-flow rate of rPET−F was measured 48.84 g/10 min, the size of rPET−F is in the range of 0.008–4 cm^3^, and the melting temperature is 245.45 °C. Ethylene glycol and antimony glycol were purchased from Sinopharm Chemical Reagent Co., Ltd. Recycled polyamide 6 pellets (rPA6) were obtained from waste PA 6 fabric by remelting and granulation, and were supplied by JING FENG Technology Co., Ltd. (Fuzhou, China).

### 2.2. Preparation of Polymer Blends and Fibers

To enhance the viscosity of rPET-F, the “alcoholysis-polymerization” strategy was used as reported in [20]. Alcoholysis extrusion of rPET-F was performed using a twin-screw extruder (MEDI-22/40, GuangZhou POTOP Co., Ltd., Guangzhou, China). rPET-F and 2 wt% ethylene glycol were mixed evenly and added into the twin-screw extruder. The screw speed of the twin-screw extruder was kept at 300 rpm, and the processing temperature was 210, 220, 230, 240, 250, 260, 268, 268, 265, 260 °C from hopper to die. Then, the alcoholysis sample and 200 ppm glycol antimony were added into the reactor (YZRJ-500M, ShangHai Tabzheng Experimental Instrument Co., Ltd., Shanghai, China), and the pressure was controlled at 0.02 MPa. The temperature was increased to 260–270 °C and maintained for 3 h to obtain the recycled polyester pellets after tackifying and crushing (rPET−T). The intrinsic viscosity of the rPET−T after tackifying is in the range of 0.72–0.74 dL/g, the melt mass-flow rate of rPET−T was measured 36.57 g/10 min, the size of rPET-T is in the range of 0.06–0.2 cm^3^, and the melting temperature is 244.43 °C.

The rPET−T was dried at 110 °C for 24 h, and rPA6 was dried under vacuum at 100 °C to avoid turning yellow. rPET-T and rPA6 were mixed by mass ratio (rPA6 varied from 2 wt% to 10 wt%), and then added into the miniature melt spinning machine (SLS-100, Wuhan Ruiming Experimental Instrument Manufacturing Co., Ltd., Wuhan, China), which is composed by miniature co-rotating twin screws. Compared with single screw, this extrusion method has a stronger plasticizing effect and can achieve better mixing in the short cavity of the micro melting spinning machine. The screw speed of the miniature melt spinning machine was kept at 50 rpm, and the processing temperature profiles were kept at 240, 260, 268, 265 °C from hopper to die. The hot draft temperature was about 100 °C, and the draft multiple was 3. The recycled fibers with different rPA6 contents (2, 4, 6, 8, 10 wt%) were prepared under the same process conditions, as shown in Table 1. The obtained recycled fibers are labeled as rPET−A_x_, where x represents the mass content of rPA6. At the same time, rPET−T and rPA6 were mixed with the same mass ratio and then added into the miniature melt spinning machine to obtain the rPET−T/rPA6 blends under the same melting process conditions without melt drafting. The blends were labeled as rPET−T/rPA6_x_; x represents the mass content of rPA6, as shown in Table 1.

### 2.3. Characterizations

The mechanical properties of the rPET−A_x_ fibers were characterized with a strength tester (CMT4104, Xinsansi Material Inspection Co., Ltd., Shenzhen, China). The pretension is 0.05 ± 0.005 cN/dtex, the distance between the two clamps is 200 mm, and the moving speed of the gripper is set as 200 mm/min. A total of 20 fibers were determined for each sample, and the average values were given to ensure accuracy and reduce experimental errors.

SEM (Regulus 8100, HITACHI, Tokyo, Japan) observation was performed to observe the phase morphology of the blends or fibers with a voltage of 5 kV. The samples for all the rPET−T/rPA6_x_ blends were placed in liquid nitrogen for 30 min, and then the samples were cryogenic ally fractured vertically to the flow direction to observe the dispersion effect of rPA6 in the matrix. The surface of the rPET−A_x_ fibers was etched with HCOOH for 12 h to remove the rPA6 phase so as to observe the distribution of rPA6 phase on the fiber surface. Before SEM observation, all the rPET−A_x_ fibers treated surfaces were cleaned with distilled water and all the samples were sputter-coated with gold.

Thermal stability performance of the rPET−A_x_ fibers was measured in an analyzer (Q50, TA Instruments, New Castle, DE, USA). 6–9 mg of rPET−A_x_ was heated from 30 to 800 °C at a heating ramp of 10 °C/min under N_2_ flow of 10 mL/min. A TA Q20 differential scanning calorimetry (DSC) instrument was used to examine the melting and crystallization of rPET-T, rPA6, and rPET−A_x_ fibers. The rPET−A_x_ fibers were sealed in aluminum hermetic pans and subjected to heat/cool/heat cycle over the temperature range 30 to 280 °C with a linear heating and cooling rate of 10 °C/min in N_2_. The glass transition temperature (T_g_), crystallization, and melting behavior of the fibers were determined from the cooling curve and second heating curve.

The rheological behavior of the rPET−A_x_ fibers was tested using a rotary rheometer (DHR-2, TA Instruments, New Castle, DE, USA) with a 25 mm ETC aluminum parallel plate. The stability of samples was checked through the dynamic time sweep test at 1 Hz and 268 °C with the stain amplitude of 1%. The rheological tests were performed at a strain of 1% (in the linear viscoelastic range) in the angular frequency range of 0.1~100 rad/s at 268 °C. The curves of complex viscosity (η), storage modulus (G′), loss modulus (G″), and loss tangent (tan δ) are obtained. The melt flow rate (MFR) of rPET-A_x_ fibers were characterized by a melt flow rate meter (MFI452-A, Shenzhen WANCE equipment Co., Ltd., Shenzhen, China), using an overhead weight of 2.16 kg at 265 °C according to GB/T 3682-2000.

^1^H-NMR was measured on a ^1^H nuclear magnetic resonance (NMR, Bruker 400 MHz, Bilerica, MA, USA) at room temperature to analyze the chemical structure at 600.1 MHz by dissolving the rPET−A_x_ fibers 10–20 mg in 1 mL of CF_3_COOD. Fourier transform infrared spectroscopy (FTIR) spectra were recorded by using a Nicoletis 10 FTIR spectrometer (Thermo Fisher Scientific Inc., Waltham, MA, USA) from 400 to 4000 cm^−1^ by averaging 32 scans at a resolution of 4 cm^−1^.

The water contact Angle tester was used to test the hydrophilicity of the rPET−A_x_ fibers surface at room temperature. The fibers were placed on the platform of the water contact Angle tester, and a drop of 4.0 μL water drop was placed on the surface of the sample to record the water contact Angle of the samples.

## 3. Results

### 3.1. Mechanical Properties of rPET-A_x_ Fibers

**Figure 1:**
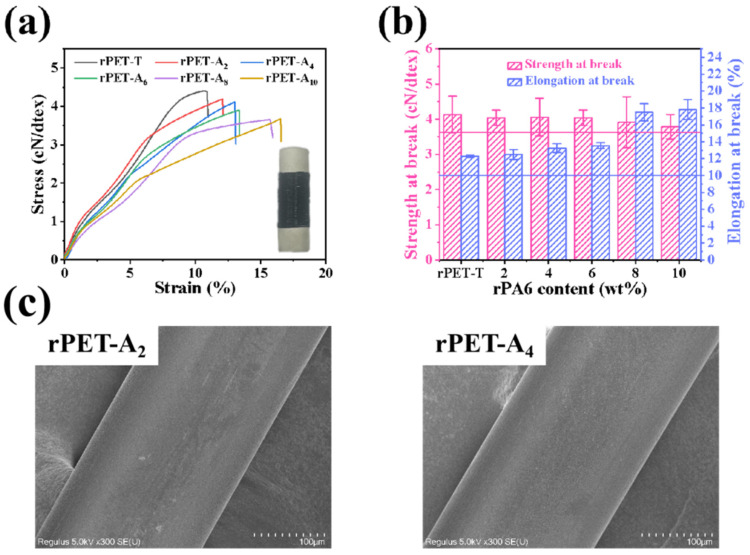
Mechanical properties of the rPET-A_x_ fibers with different rPA6 content. (**a**) Stress-strain curves, (**b**) Mechanical properties statistical chart, and (**c**) morphology diagram.

Compared with virgin PET particles, wasted polyester fabrics contain impurities such as spinning oils, dyes, and other fibers that are difficult to separate. The intrinsic viscosity of rPET-T obtained by in-situ reaction can be significantly improved, maintaining at 0.72–0.74 dL/g, thereby meeting the needs of spinning [21]. The DSC, TGA, FTIR, and rheological curves are shown in Figure S1. By adjusting the processing temperature and drafting speed of the spinning machine, rPET-A_x_ fibers (x = 2, 4, 6, 8, 10) with a stable linear density at 50–200 dtex can be obtained. The microscopic morphology of the rPET-A_x_ fibers is shown in Figure 1c, using rPET-A_2_ and rPET-A_4_ as example. Tensile stress-strain curves and mechanical properties of the rPET-A_x_ fibers are shown in Figure 1. The results show that the strength at break decreased from 4.13 cN/dtex for the rPET-T fibers to 4.04 cN/dtex for the rPET-A_2_ fibers, a total decrease of 2.18%. The elongation at break increased from 12.28% for the rPET-T fibers to 12.50%, a total increase of 1.79%. Thus, the existence of 2 wt% rPA6 reduces the breaking strength and improves the elongation at break of recycled fibers. As the content of rPA6 increased continuously, the strength at break decreased to 3.91 cN/dtex for rPET-A_8_ fibers and 3.79 cN/dtex for rPET-A_10_ fibers, a total decrease of 8.23%. The elongation at break decreased to 17.48% for rPET-A_8_ fibers and 17.19% for rPET-A_10_ fibers, a total increase of 44.87%. The strength at break of the recycled fibers decreased, and the elongation at break increased continuously.

According to the industry standard (FZ/T 54127-2020), the strength at break of recycled polyester fiber superior products is required ≥3.6 cN/dtex, and the elongation at break ≥10%, as shown in the indicator line in the figure. The mechanical properties of rPET-A_x_ fibers were tested in compliance with the operational requirements of the standard (GB/T 14344-2022). When the rPA6 content is less than 10 wt%, the mechanical properties of the recycled fibers are suitable for applications in the textile field.

### 3.2. Hydrophilic Properties of the rPET-A_x_ Fibers

**Figure 2:**
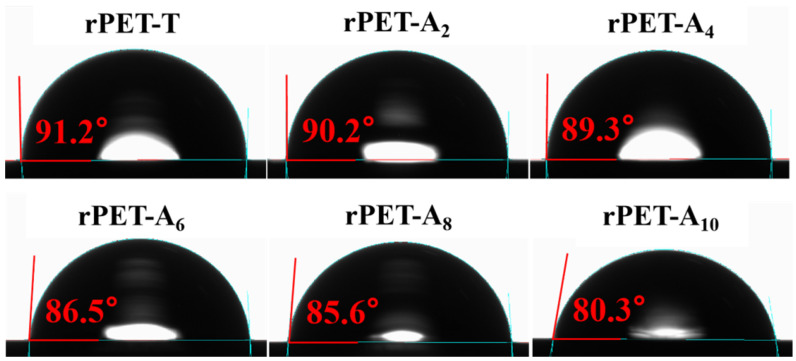
Contact Angle of regenerated fiber with different rPA6 content.

Compared with PET fiber, PA6 fiber has better hydrophilic properties. On this basis, the hydrophilic properties of the rPET-A_x_ fibers were investigated. As shown in Figure 2, the contact angle of rPET-A_x_ fibers decreases from 91.3° to 80.3° for the rPET-A_10_ with the increasing proportion of rPA6. Therefore, the hydrophilic performance of recycled fibers are improved by blending with rPA6.

**Figure 3:**
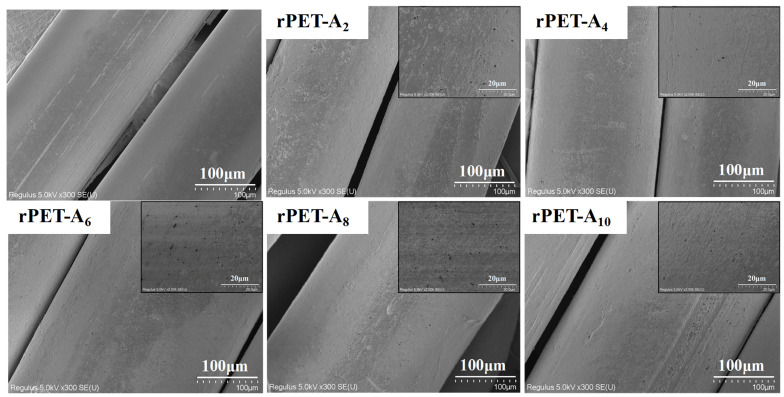
Micromorphology of the rPET-A_x_ fibers with different rPA6 content.

Figure 3 shows the morphology of the rPET-A_x_ fibers surface before and after formic acid (FA) etching for 12 h. Due to the solubility of PA6 in FA, the rPA6 phase in the surface was removed after etching. Figure 1c shows the micro-morphology of rPET-A_2_ and rPET-A_4_ fibers without etching. It can be found that the fiber surface is smooth and flat without etching. After etching, spherical holes appeared on the fiber surface, which indicates that the rPA6 was evenly dispersed on the rPET-A_x_ fiber surface. PA6 and PET are incompatible materials based on previous reports; the uniform dispersion is probably due to the ester-amide exchange reaction.

### 3.3. Phase Morpchology of rPET-T/rPA6 Blends

**Figure 4:**
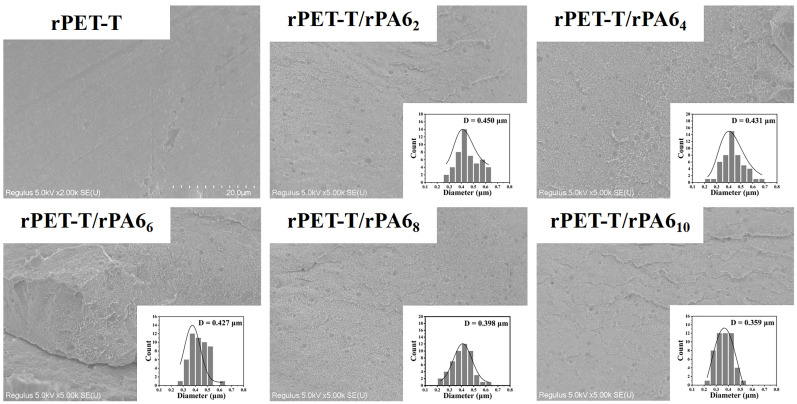
Phase morphology of the rPET-T/rPA6_x_ samples with different rPA6 content and the inset present the histogram for domain size distribution of the rPA6.

The mechanical properties of the recycled fibers are highly related to the compatibility of the two phases [22,23]. Therefore, we collected the rPET-T/rPA6_x_ samples, which were obtained under the same melt processing conditions as rPET-A_x_ fibers (prepared without drawing), and then observed its micro-morphology by brittle fracture. The cross-section SEM images of the rPET-T/rPA6_x_ are shown in Figure 4. A typical sea-island morphology structure was observed; there are no cracks or separations in the observation area, indicating that there is no obvious fracture or deformation phenomenon after the extrusion of rPET-T and rPA6. With the increase of rPA6 content, the average size of rPA6 dispersed phase gradually decreased. The average size of rPA6 gradually decreased from 0.450 μm for rPET-T/rPA6_2_ to 0.359 μm for rPET-T/rPA6_10_, which is much smaller than that of the incompatible two-phase system mentioned in other studies [24,25,26]. It indicated that rPA6 was well dispersed in the rPET-T matrix.

### 3.4. The Thermal and Crystallization Properties of rPET-A_x_ Fibers

**Figure 5:**
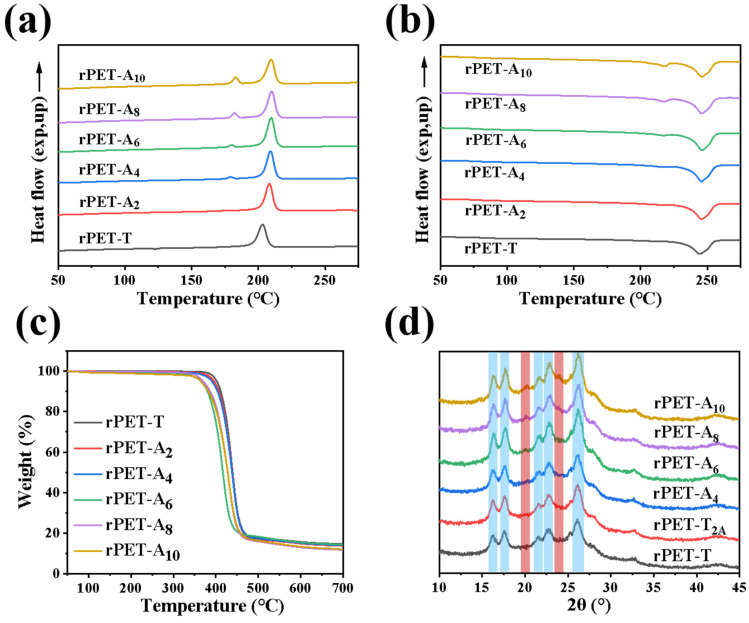
Thermal properties and crystal structures of the rPET-A_x_ fibers: DSC curves of rPET-Ax fibers: (**a**) cooling process, (**b**) melting process; (**c**) TGA curves of rPET-A_x_ fibers, and (**d**) XRD curves.

Thermal and crystallization properties have important significance for the processing and properties of materials [27]. The effects of rPA6 on the thermal properties and crystallization behavior of the rPET-A_x_ fibers were discussed. Figure 5a,b show DSC curves of the rPET-T and the rPET-A_x_ fibers, respectively. There are two crystallization and melting peaks in the first cooling curve (Figure 5a) and the second heating curve (Figure 5b). Detailed calorimetric data of DSC curves are shown in Table S2. The melting peak around 245 °C and crystallization peak around 205 °C correspond to the melting and crystallization of the rPET-T phase in the rPET-A_x_ fibers; the melt peak around 205 °C and crystallization peak around 180 °C corresponds to the rPA6 phase. The melting temperature (T_m_) of the rPET-T phase maintains stability. The crystallization temperature (T_c_) of the rPET-T phase increases from 203.08 °C to 208.26 °C after blending with 2 wt% rPA6. With the increasing proportion of rPA6 content, the T_m_ of the recycled fibers maintains stability; the T_c_ no longer increases and stabilizes around 208 °C. The T_m_ of the rPA6 phase is around 217 °C; the T_c_ of the rPA6 phase decreases from 174.91 °C for the rPET-A_2_ fiber to 182.90 °C for the rPET-A_10_.

The crystallinity degree (*X*_c_) of the recycled fibers increased to 28.06% for the rPET-A_2_ fibers. The crystallinity of the rPET-A_x_ fibers remained around 28.50% when the content of rPA6 ranged from 4 to 10 wt%. This is probably because the ester amide exchange reaction improves the compatibility of rPET-T/rPA6 blends, which results in a decrease in the grain size of rPET-T. Heterogeneous nuclei are easily formed at both the copolymers and interface of rPET-T and rPA6, so that PET phase can crystallize at a higher temperature [28]. The melting peak of rPA6 appears to be an acromial phenomenon when the proportion of rPA6 components increases to 6 wt%. The appearance of acromion indicates that the crystallization process of rPA6 is influenced by PET crystals, which leads to the appearance of imperfect crystals [29]. As a result, the crystallinity of rPA6 is improved. The rPET-T as a heterogeneous nucleating agent; it promoted the crystallization of rPA6 and increased the crystallinity and crystallization temperature at the same time. PET and PA6 are incompatible two-phase systems; although the copolymer produced by the ester amide exchange reaction improves the compatibility, the partial compatibility of the two phases still leads to the decrease of the strength of break. Despite this, a small amount of PA 6 with good flexibility in the fiber promoted the movement of PET segments during the stretching process, so the elongation at break increased.

Figure 5c shows the thermogravimetric curves of the rPET-A_x_ fibers. The thermal decomposition temperature (T_d_) of the rPET-A_x_ fibers decreased from 402.22 °C for the rPET-T to 396.06 °C for the rPET-A_2_, decreased to 364.77 °C for the rPET-A_10_, and the thermal stability of the rPET-A_x_ fibers obviously decreased with the increase of the rPA6 content. Figure 5d shows the XRD pattern of rPET-T and the rPET-A_x_ fibers. rPET-T has obvious characteristic diffraction peaks located at 16.4°, 17.7°, 21.5°, 22.8°, and 26.1°, as shown in the blue area. The three main peaks (2θ = 17.7, 22.8°, 26.1°) are assigned to the (010), (110), and (100) planes of the triclinic structure, respectively. The characteristic diffraction peaks located at 20.2° and 23.9° are assigned to the (020) and (200) planes of the rPA6 α crystal, respectively, as shown in the red area. Compared with rPET-T and rPA6, the location of the characteristic diffraction peaks does not change significantly. With the increase of rPA6 content, the characteristic peak of the blend at 17.7, 22.8, and 26.1° is enhanced; this is the triclinic structure of rPET-T. The peaks at 20.2° and 23.9° are the α crystal (200) crystal plane of rPA6. The crystallinity of the recycled fibers calculated from the XRD pattern increases as the rPA6 content increases, which is consistent with the result of DSC as shown in Table S1.

Therefore, when there is a small amount of rPA6, the crystallization temperature and the crystallinity of the two phases increases. Both the copolymers and the interface of rPET-T and rPA6 appear as heterogeneous nuclei on the crystallization of PET phase in the blend. The above-mentioned analysis results show that the existence of rPA6 changes the crystallization behavior and thermal stability of the recycled fibers, which helps to find suitable processing temperatures for the rPET-T/rPA6 blend.

### 3.5. Rheological Properties of rPET-A_x_ Fibers Melts

**Figure 6:**
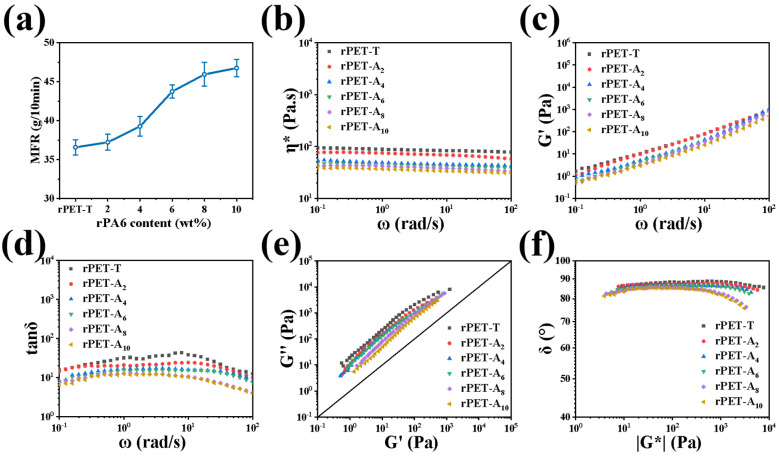
Rheological properties of the rPET-T and the rPET-A_x_ fibers with different rPA6 content at 265 °C: (**a**) MFR curves, (**b**) complex viscosity, (**c**) storage modulus, (**d**) loss tangent, (tan δ) on frequency, (**e**) Han plots, and (**f**) vGP plots. (Where ω stands for angular velocity, G′ stands for Loss modulus, and G* stands for complex modulus).

In this section, the properties of rPET-A_x_ fibers were investigated using a rotary rheometer. The MFR curves and rheological properties of rPET-A_x_ fibers are shown in the Figure 6. As shown in Figure 6a, with the increase of the rPA6 content, the MFR of the rPET-A_x_ fibers melt increased from 36.57 g/10 min for the pure rPET-T to 46.75 g/10 min for the rPET-A_10_. Figure 6b shows the complex viscosity of the rPET-A_x_ fibers at 268 °C. The existence of rPA6 reduces the complex viscosity, which is consistent with the MFR results. The result show that the rPA6 significantly improves the fluidity of the melt. Both the G′ and G″ of the rPET-A_x_ fibers decreases with the increase of rPA6 content at full shear rates. This is because of the lower viscosity of rPA6. These results show that rPA6 can decrease the resistance of the melt molecular chain movement and decrease the movement of the chain segment hysteresis, thereby promoting the draft of the melt [30].

The loss factor is a significant indicator reflecting the viscosity and elasticity of the melt [31,32]. As can be seen in Figure 6d, the loss factor of the melt decreases with the increase of rPA6 content, and the elasticity of the melt increases. Moreover, it is found that with the increase of rPA6 content, the curves gradually shift to the right, and with the increase of frequency range, the change amplitude of G′ is slightly higher than that of G″, as seen in Figure 6e. The blend gradually approaches the isomodulus line with the increase of rPA6 content, the Han curves of blends are consistent, and the fibers of different content maintain a good linear relationship within a certain test frequency range [33]. The result shows that the melt behavior changes from liquid to solid after blending and the melt elasticity is enhanced, which means that the strength of melt is also improved [34]. The results above mentioned suggest that the chemical reaction between rPET-T and rPA6 may have changed the structure of some of the molecules. To characterize whether the changes in the chain segment structure caused by this chemical reaction will affect the rheological properties of the polymer melt, the vGP diagram of the rPET-A_x_ fibers was obtained through rheological data, as shown in Figure 6f. It can be seen that the curves of rPET-T, rPET-A_2_, rPET-A_4_, and rPET-A_6_ fibers did not change much with the increase of |G*|. It can be approximated that the chain segment structure of the blended melt was mainly a linear chain, like that of rPET-T [35]. The difference is that the δ value of rPET-A_8_ fibers and rPET-A_10_ fibers in the high value region have a more obvious trend of decline. The δ value of the blend remained above 80° in both the low and the median regions, and the material was mainly viscous.

### 3.6. Chemical Structure of rPET−A_x_ Fibers

**Figure 7:**
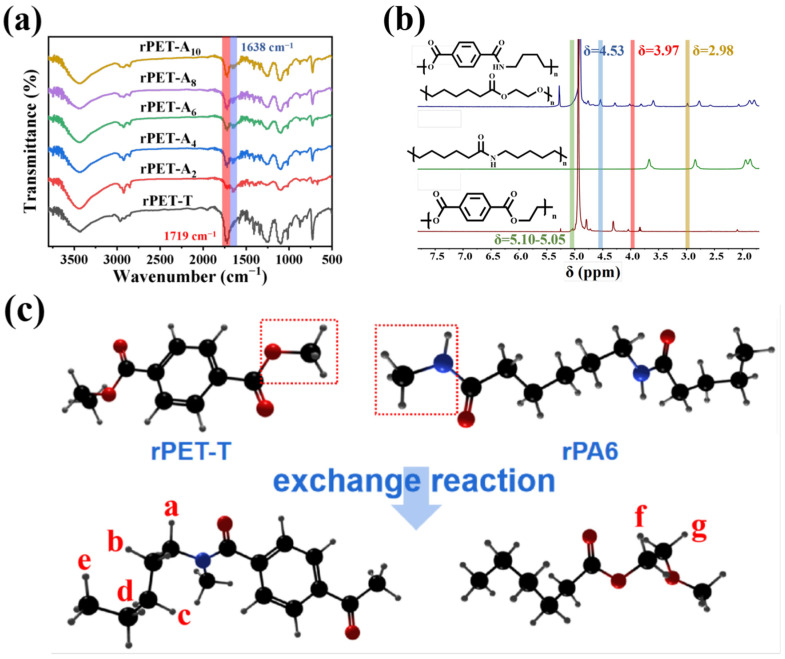
FTIR spectra (**a**) ^1^H NMR, (**b**) Reaction diagram, (**c**) of the rPET-A_x_ fibers.

To investigate the chemical structural changes between rPET-T, rPA6, and rPET-A_x_, the FT-IR spectra and ^1^H NMR spectra are shown in Figure 7. The stretching vibration peak of the C=O bond and the aromatic ring skeleton are located at 1719 cm^−1^ and 1605 cm^−1^, respectively, while the deformation vibration peak of the C-H bond on the benzene ring is at 873 cm^−1^ and the out-of-plane bending vibration peak of the carbonyl group is at 729 cm^−1^. These characteristic peaks prove the chemical structure of the rPET-T chain. The FT-IR spectrum of rPA6 is shown in Figure S2a. There are in-plane deformation vibrations and frequency doubling peaks of N-H at 1542 cm^−1^ and 3084 cm^−1^, respectively, and a C=O amide I band and a C-C (O) stretching vibration peak located at 1638 cm^−1^ and 1168 cm^−1^, respectively. The peak at 1638 cm^−1^ proves the existence of rPA6 in the rPET-A_x_ fibers. Infrared spectroscopy can qualitatively analyze the functional group structure of the molecular chain of the blend. The functional group changes caused by the self-compatible copolymer produced by the segment exchange reaction should be observed. Therefore, the ^1^H NMR spectra of the rPET-T, rPA6, and rPET-A_10_ in CF_3_COOD were measured, as shown in Figure 7b. The chemical shift peaks of the H in benzene ring of rPET-T chain located at 8.22 ppm and peak of H in methylene at 4.88 ppm. The chemical shift peak at 3.92 ppm is H on C closest to N-H and peak at 3.10 ppm is H on C closest to C=O. The remaining H corresponds to the chemical shift at 2.20, 2.11, and 1.87 ppm. It can be seen from Figure 7b that the chemical shifts at positions H of the rPET-T chain segments a and b correspond to 4.31 ppm and 2.10 ppm, as shown in the red area. After blending with rPA6, the ester amide exchange reaction occurred in part of the chain segment, as shown in Figure 7c. Because the amide group has a weaker electron absorption ability than the ester group, H at the position a and b will move towards the higher field after the ester bond of rPET-T received the rA6 chain segment; this is the chemical shift at 2.98 ppm and 1.54 ppm, as shown in the blue area.

**Scheme 1:**
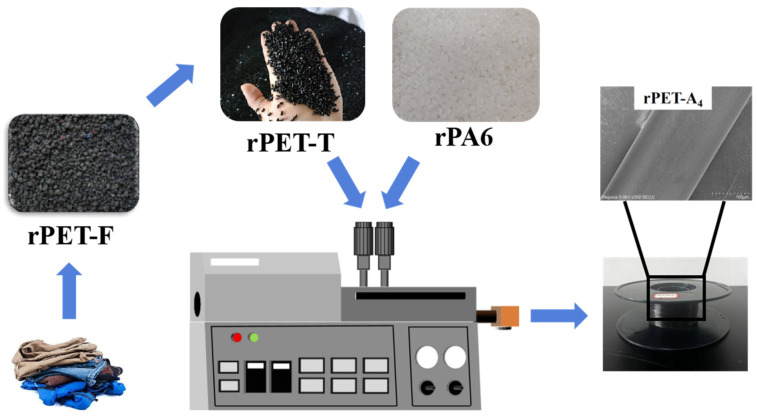
Schematic diagram of recycled fiber preparation by in-situ reaction modification of polyester and nylon.

The good compatibility of rPA6 and rPET-T may be attributed to the occurrence of exchange reaction and the obtained copolymers as compatibilizer. The principle is concluded as shown in Scheme 1. When a small amount of rPA6 is blended with PET, the resulting self-compatible copolymer can improve the compatibility of a small amount of PET with PA6. The chemical reaction and obtained copolymers play the most critical role in improving the compatibility of the two phases.

## 4. Conclusions

In this work, the influence of the presence of a small amount rPA6 on the properties of recycled fibers was systematically discussed. The results show that when there is a small amount of rPA6 (≤10 wt%) in the polyester melt, the self-compatible copolymer obtained through the chemical reaction between ester and amide can improve the compatibility between rPET-T and rPA6, so the rPA6 can be well dispersed in the PET matrix. It also increases the crystallization capacity of the polymer and the copolymer, and the interface between rPET-T and rPA6 acts as heterogeneous nucleus, which increases the crystallization temperature of the blend. Due to the influence of rPA6, the complex viscosity of the rPET-A_x_ fibers is decreased but remains at a high level. The complex viscosity changes little in the high angular frequency and the melt strength remains stable, which is expected to achieve long-term stable spinning of the melt.

In the process of melt spinning, rPA6 can be well dispersed on the surface of the rPET-A_x_ fiber, so the hydrophilicity also improved. Under the combined action, the strength at break of the rPET-A_x_ fibers decreases and the elongation at break increases. When the rPA6 content is less than 10 wt%, the strength at break of the rPET-A_x_ fibers is greater than 3.0 cN/dtex, and the elongation at break is increased from 12% to 18%. All the indexes meet the requirements of recycled fiber products.

## Figures and Tables

**Table 1:** The formulation of rPET-A_X_ fibers and rPET-T/rPA6_x_ blends.

Sample	rPET-T(wt%)	rPA6(wt%)	Drafting Condition
rPET−A_2_	98	2	Hot draft temperature: 100 °C Draft multiple: 3
rPET−A_4_	96	4	
rPET−A_6_	94	6	
rPET−A_8_	92	8	
rPET−A_10_	90	10	
rPET-T/rPA6_2_	98	2	Without drafting
rPET-T/rPA6_4_	96	4	
rPET-T/rPA6_6_	94	6	
rPET-T/rPA6_8_	92	8	
rPET-T/rPA6_10_	90	10	

## Data Availability

Date are contained within the article and Supplementary Materials.

## Supplementary Materials

The following supporting information can be downloaded at: https://www.mdpi.com/article/10.3390/polym16081052/s1, Figure S1: The infrared spectrum (a) and complex viscosity against angular frequency (b) of polyester raw materials; Figure S2: Infrared spectrum (a), thermogravimetric curve (b), DSC curve (c), complex viscosity to angular frequency mapping (d) of waste nylon fiber; Table S1: Glass transition temperature (T_g_), crystallization temperature (T_c_), melting temperature (T_m_), crystallinity and thermal decomposition temperature (T_d_) of waste nylon; Table S2: Thermal performance data of rPET-A_x_.
