# Supplementary material for: Recycled PET/PA6 Fibers from Waste Textile with Improved Hydrophilicity by In-Situ Reaction-Induced Capacity Enhancement
Source: Polymers (Basel). 2024 Apr 11;16(8):1052. doi: 10.3390/polym16081052 (PMC11054667; doi:10.3390/polym16081052)
Supplement: Supplementary file 1 [file polymers-16-01052-s001.zip › polymers-2926049-supplementary.pdf]

Supplementary Material

Recycled PET/PA6 Fibers from Waste Textile with Improved Hydrophilicity by In-Situ Reaction-Induced Capacity Enhancement

1. The preparation of rPET-T

Before the experiment, the waste fiber source polyester foam (rPET-F) was placed in a blast drying oven at 120 °C for 12 h, and then mixed with 2wt% ethylene glycol and put into the twin screw for melting and extrusion. The temperature of the main screw plasticizing section of the twin screw extruder was set at 268°C. The alcoholysis sample was evenly mixed with 200 ppm glycol antimony and put into the reactor for polycondensation reaction. The temperature of the reactor was set at 268°C, the reaction vacuum was ≤200 Pa, and the reaction time was 3h. During the reaction process, the viscosity of the polyester melt was increased to obtain recycled polyester particle (rPET-T).

2. Physical and chemical properties of rPET-T

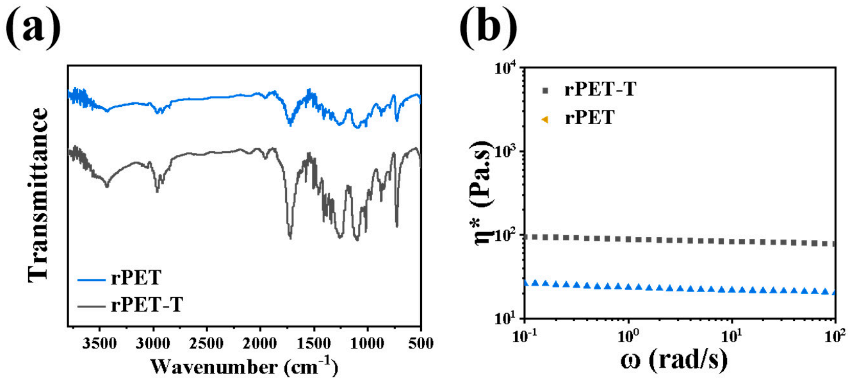

Figure S1. The infrared spectrum(a) and complex viscosity against angular frequency(b) of polyester raw materials

Figure S1(a) shows the presence of aromatic rings at 721 cm^{-1}, 871 cm^{-1} and 1238 cm^{-1}, the presence of hydroxyl (-OH) at 3100 cm^{-1} and 3500 cm^{-1}, and the presence of methylene oxide ($-OCH_2$) at 1116 cm^{-1}. It can be seen from the figure that the infrared spectra of the two polyesters have no significant difference in absorption peaks at the above positions, and the chemical structure is consistent. Figure S2 (b) is a plot of the complex viscosity of the two polyesters as a function of angular frequency. It can be seen from the figure that the complex viscosity of rPET and RPET-T are basically maintained in the same platform in the whole frequency range, which indicates that the viscosity stability of PET is good, and the molecular structure is a linear chain. It can also be found that the complex viscosity of rPET-F is lower throughout the frequency range, and the molecular weight of rPET-F is lower.

3. Physical and chemical properties of rPA6

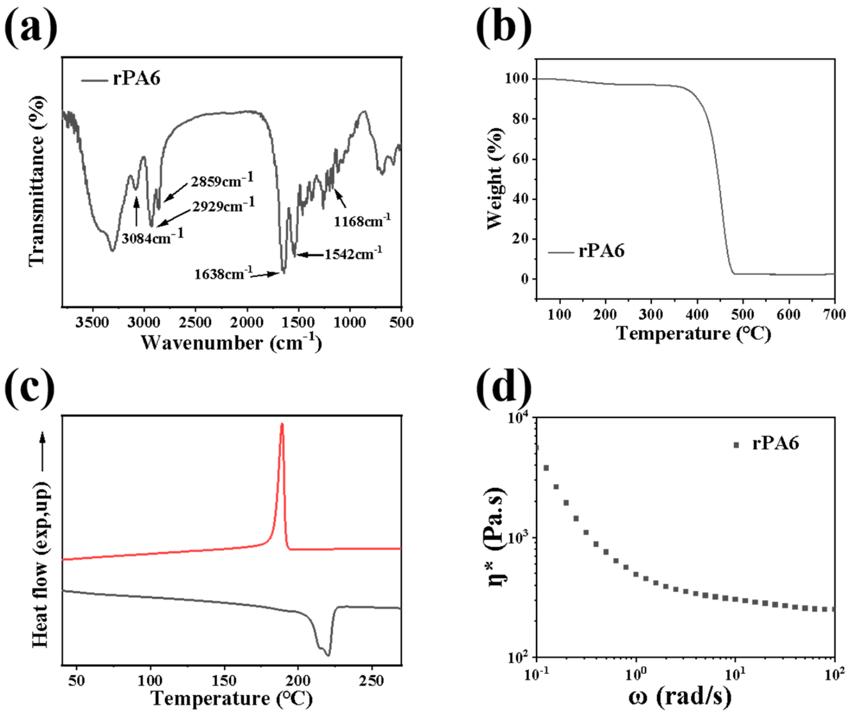

Figure S2. Infrared spectrum (a), thermogravimetric curve (b), DSC curve (c), complex viscosity to angular frequency mapping (d) of waste nylon fiber

Table S1. Glass transition temperature (T_g), crystallization temperature (T_c), melting temperature (T_m), crystallinity and thermal decomposition temperature (T_d) of waste nylon

Sample	T_g (°C)	T_c (°C)	T_m (°C)	T_d (°C)
rPA6	57.26	188.64	220.74	378.75

Figure S2(a) is the infrared spectrum of waste nylon. It can be observed from the figure that there are N-H deformation vibration and frequency doubling peaks of deformation vibration in rPA6 at 1542cm^{-1} and 3084cm^{-1}, and methylene antisymmetric and stretching vibration peaks at 2929cm^{-1} and 2859cm^{-1}. At 1638cm^{-1}, there is a C=O stretching vibration with amide I band, N-H deformation vibration at 1542cm^{-1}, and C-C(O) stretching vibration peak at 1168cm^{-1}.

Figure S2(b) and (c) are the thermogravimetric curves and the rise and fall curves of DSC respectively. Table S2 is the thermal performance data of waste nylon fiber. It can be seen from the chart that T_g of rPA6 is 57.26°C, T_c is 188.64°C, T_m is 220.74°C, and T_d is 378.75°C. Figure S2 (d) is the rheological curve of waste nylon. It can be seen that the complex viscosity of rPA6 in the low frequency region decreases with the increase of angular velocity. At a relatively low shear rate, the complex viscosity decreases greatly; at a relatively high shear rate, the complex viscosity decreases slowly, showing an obvious pseudoplastic fluid characteristic of "thinning shear force".

4. Thermal performance data of rPET-A_x

Table S2 Thermal performance data of rPET-A_x

	T_g(°C)	T_{c1}(°C)	T_{c2}(°C)	T_{m1}(°C)	T_{m2}(°C)	T_d(°C)	X_{c1}(%)	X_{c2}(%)
rPET-T	80.18		203.08		244.43	402.22	-	27.54
rPET-A_2	78.25	174.91	208.26	217.21	245.85	396.06	-	28.38
rPET-A_4	76.30	178.82	209.14	217.66	245.83	390.81	20.48	28.45
rPET-A_6	76.18	179.30	209.60	217.18	246.15	364.77	21.18	28.85
rPET-A_8	76.87	182.02	210.05	217.51	245.96	369.91	25.63	28.61
rPET-A_{10}	76.44	182.90	209.55	217.93	243.98	364.77	27.23	28.43

1 T_{C1} is the crystallization temperature of rPA6 phase in the blend

2 T_{C2} is the crystallization temperature of rPET-T phase in the blend

3 T_{m1} is the Melting temperature temperature of rPA6 phase in the blend

4 T_{m2} is the Melting temperature temperature of rPET-T phase in the blend

5 X_{C1} is the crystallinity of rPA6 phase in the blend

6 X_{C2} is the crystallinity of rPET-T phase in the blend
